# New insights about phenotypic heterogeneity within *Propionibacterium freudenreichii* argue against its division into subspecies

**DOI:** 10.1007/s13594-015-0229-2

**Published:** 2015-05-08

**Authors:** Rosangela de Freitas, Marie-Noelle Madec, Victoria Chuat, Marie-Bernadette Maillard, María C. Abeijón Mukdsi, Hélène Falentin, Antonio Fernandes de Carvalho, Florence Valence, Anne Thierry

**Affiliations:** 1 Departamento de Tecnologia de Alimentos, Universidade Federal de Viçosa, Viçosa, MG Brazil; 2 INRA, UMR1253 Science et Technologie du Lait et de l‘OEuf, 35042 Rennes, France; 3 AGROCAMPUS OUEST, UMR1253 Science et Technologie du Lait et de l‘OEuf, 35042 Rennes, France; 4 Present Address: Centro de Referencia para Lactobacilos (CERELA-CONICET), Chacabuco 145, (4000), Tucumán, Argentina

**Keywords:** Propionibacteria, Subspecies, Biodiversity, Volatile fingerprint, Flavour compound

## Abstract

*Propionibacterium freudenreichii* is widely used in Swiss-type cheese manufacture, where it contributes to flavour and eye development. It is currently divided into two subspecies, according to the phenotype for lactose fermentation and nitrate reduction (lac^+^/nit^−^ and lac^−^/nit^+^ for *P. freudenreichii* subsp. *shermanii* and subsp. *freudenreichii*, respectively). However, the existence of unclassifiable strains (lac^+^/nit^+^ and lac^−^/nit^−^) has also been reported. The aim of this study was to revisit the relevance of the subdivision of *P. freudenreichii* into subspecies, by confirming the existence of unclassifiable strains. Relevant conditions to test the ability of *P. freudenreichii* for lactose fermentation and nitrate reduction were first determined, by using 10 sequenced strains, in which the presence or absence of the lactose and nitrate genomic islands were known. We also determined whether the subdivision based on lac/nit phenotype was related to other phenotypic properties of interest in cheese manufacture, in this case, the production of aroma compounds, analysed by gas chromatography-mass spectrometry, for a total of 28 strains. The results showed that a too short incubation time can lead to false negative for lactose fermentation and nitrate reduction. They confirmed the existence of four lac/nit phenotypes instead of the two expected, thus leading to 13 unclassifiable strains out of the 28 characterized (7 lac^+^/nit^+^ and 6 lac^−^/nit^−^). The production of the 15 aroma compounds detected in all cultures varied more within a lac/nit phenotype (up to 20 times) than between them. Taken together, these results demonstrate that the division of *P. freudenreichii* into two subspecies does not appear to be relevant.

## Introduction

*Propionibacterium freudenreichii* is the main dairy propionibacteria species used as secondary culture in cheese manufacture, in particular for Swiss-type cheeses (Cummins and Johnson 1986). It produces varied aroma compound in cheese from lactate fermentation, amino acid catabolism, and milk fat hydrolysis, and CO_2_. These products significantly contribute to the development of the typical flavour and the formation of eyes in Swiss-type cheeses (Thierry et al. 2011, Abeijon Mukdsi et al. 2014). The production of aroma compounds is highly strain-dependent (Yee et al. 2014). Moreover, *P. freudenreichii* has potentials as bioprotective cultures by producing antimicrobial compounds such as bacteriocins and organic acids (Tharmaraj and Shah 2009) and antifungal peptides (Jan et al. 2007). It is also able to produce a variety of beneficial compounds for human health, such as vitamin B12 and folic acid (Hugenholtz et al. 2002), and some strains have been proposed as probiotics, due to their ability to modulate intestinal microbiota through their bifidogenic effect and to anti-inflammatory effects and antimutagenic properties, among others (Cousin et al. 2011).

*P. freudenreichii* is currently divided into two subspecies, on the basis of two phenotypical criteria (Cummins and Johnson 1986). *P. freudenreichii* subsp. *freudenreichii* does not ferment lactose and possess a nitrate reductase activity (lac^−^/nit^+^), whereas it is the opposite for *P. freudenreichii* subsp. *shermanii* (lac^+^/nit^−^). However, the existence of strains harbouring the two other possible phenotypes (lac^+^/nit^+^ or lac^−^/nit^−^) has occasionally been mentioned (de Carvalho et al. 1994; Kaspar 1982; Moore and Holdeman 1974; Vorobjeva 1999). More recently, in a study which characterized a collection of 113 strains of *P. freudenreichii*, more than 25% of strains could not be assigned to a subspecies, and were labelled “pheno+” (lac^+^/nit^+^) or “pheno^−^” (lac^−^/nit^−^) (Dalmasso et al. 2011). The ability to ferment lactose and reduce nitrate results from the presence of genomic islands (Falentin et al. 2010; Loux et al. 2015). Regarding the use of *P. freudenreichii* for cheese ripening, the ability to ferment lactose can be an important criterion in some cheese varieties containing residual lactose at the beginning of the ripening, therefore resulting in the choice of strains of the subspecies *P. freudenreichii* subsp. *shermanii*. However, strains harbouring the phenotype lac^+^/nit^+^ could be also chosen in this case. Besides, the possible relationships between the subdivision of the species and other technologically important properties are unknown.

Molecular methods have failed up to date to distinguish *P. freudenreichii* subspecies (Dasen et al. 1998; Fessler et al. 1999; Tilsala-Timisjarvi and Alatossava 2001), and therefore phenotypic methods are still required. Some discrepancies between studies could also have been due to a lack of standardization of phenotypical tests, since it is known that experimental conditions such as the presence of O_2_, temperature, and incubation time can influence the results observed (Busse et al. 1996; Swart et al. 1998).

The aim of this study was to revisit the relevance of the subdivision of *P. freudenreichii* into subspecies, by confirming the existence of unclassifiable strains. For this, the most relevant conditions to test *P. freudenreichii* for its activity of lactose fermentation and nitrate reduction were first determined by using a set of sequenced strains for which the expected phenotype could be predicted from their genotype. In addition, the possible relationships between the subspecies and the production of some important aroma compounds were investigated.

## Materials and methods

### Strains and culture conditions

A total of 28 strains of *P. freudenreichii* were used, including 10 previously sequenced (Loux et al. 2015) strains provided by the International Centre for Microbial Resources collection of bacteria (CIRM-BIA, UMR1253, INRA Rennes, France) and 18 Brazilian strains (Table 1). The genomic data of sequenced strains were analysed using the AGMIAL platform (Bryson et al. 2006). The Brazilian strains were isolated from dairy farms situated in Minas Gerais (Freitas et al. 2015). Before phenotypical characterization, the strains were reactivated from frozen (−80 °C) glycerol (Grosseron, Saint-Herblain, France) stocks and grown in yeast extract lactate (YEL) medium (Malik et al. 1968) incubated at 30 °C for 48 h.

**Table 1:** Results of phenotypical tests of lactose fermentation and nitrate reduction evaluated after 2, 5, and 7 days of incubation, and presence or absence of lactose and nitrate reductase genomic islands, *lac* GI and *nar* GI, respectively

Strain	Strain origin		Lactose			Nitrate		Phenotype^a^	*lac* GI^b^	*nar* GI^b^
	Biotope	Country	2 days	5 days	7 days	2 days	5 days			
CIRM-BIA121^T c^	Swiss cheese	USA	0	0	0	1	1	F	0	1
CIRM-BIA514	Hay	France	0	0	0	1	nd	F	0	1
CIRM-BIA9	Emmental cheese	The Netherlands	0	1	1	0	0	S	1	0
CIRM-BIA118	Gruyère cheese	France	0	1	1	0	0	S	1	0
CIRM-BIA135	Ewe raw milk	France	0	0	1	0	0	S	1	0
CIRM-BIA508	Yack cheese	Nepal	0	1	1	0	0	S	1	0
CIRM-BIA1^T^	unknown	Unknown	0	1	1	0	0	S	1	0
CIRM-BIA122	unknown	Unknown	0	1	1	0	1	P+	1	1
CIRM-BIA513	Gruyère cheese	France	0	1	1	0	1	P+	1	1
CIRM-BIA516	Ras cheese	Egypt	0	0	1	1	nd	P+	1	1
B44	Milk	Brazil	0	0	0	0	1	F	nd	nd
B46	Milk	Brazil	0	0	0	0	1	F	nd	nd
B158	Soil	Brazil	0	0	0	1	nd	F	nd	nd
B70	Milk	Brazil	1	nd	nd	0	0	S	nd	nd
B78	Milk	Brazil	0	1	1	0	0	S	nd	nd
B86	Soil	Brazil	1	nd	nd	0	0	S	nd	nd
B91	Silage	Brazil	1	nd	nd	0	0	S	nd	nd
B141	Milk	Brazil	0	1	1	0	0	S	nd	nd
B42	Milk	Brazil	0	1	1	1	nd	P+	nd	nd
B43	Milk	Brazil	1	nd	nd	0	1	P+	nd	nd
B66	Grass	Brazil	1	nd	nd	0	1	P+	nd	nd
B82	Soil	Brazil	0	1	1	1	nd	P+	nd	nd
B75	Milk	Brazil	0	0	0	0	0	P−	nd	nd
B89	Soil	Brazil	0	0	0	0	0	P−	nd	nd
B145	Milk	Brazil	0	0	0	0	0	P−	nd	nd
B148	Milk	Brazil	0	0	0	0	0	P−	nd	nd
B171	Grass	Brazil	0	0	0	0	0	P−	nd	nd
B172	Grass	Brazil	0	0	0	0	0	P−	nd	nd

*nd* not determined

^a^Phenotype: *freudenreichii* (F), *shermanii* (S), lac^+^/nit^+^ (P+) and lac^−^/nit^−^ (P−)

^b^From the genome sequence (Loux et al. 2015)

^c^T, type strain

### Lactose fermentation and nitrate reduction

Two concentrations of lactose and potassium nitrate were tested for a pool of strains in preliminary tests.

Lactose fermentation was tested in a modified API 50CH medium containing the following: lactose 5 or 20 g.L^−1^ (Panreac, Lyon, France), tryptone 10 g.L^−1^, yeast extract 5 g.L^−1^, K_2_HPO_4_, 0.25 g.L^−1^, MnSO_4_ 0.05 g.L^−1^, and bromocresol purple 0.17 g.L^−1^. The medium was inoculated using 1% (*v*/*v*) of 48-h cultures in YEL, and incubated at 30 °C under anaerobiosis (using the Anaerocult A system, Merck, Darmstadt, Germany). The production of acid from lactose was determined from the colour change of bromocresol purple from purple to yellow after 2, 5, and 7 days of incubation.

Nitrate reductase activity was detected by means of the Griess reagent (Biomérieux, Marcy l'Etoile, France) after incubation of cultures at 30 °C under microaerophilic conditions (air atmosphere without agitation) in a broth containing potassium nitrate, 0.5 or 1.5 g.L^−1^ (VWR International, Fontenay-sous-Bois, France), tryptone (Biokar Diagnostics, Allone, France) 10 g.L^−1^, yeast extract (Biokar Diagnostics) 5 g.L^−1^, and glucose (Grosseron, Saint-Herblain, France) 1 g.L^−1^, according to Dalmasso et al. (2011). The results of the tests were read after 2 and 5 days of incubation.

All the tests were carried out in triplicate.

### Analysis of aroma compounds

To test strains for their ability to produce aroma compounds, cultures were grown in YEL supplemented by ethanol (final concentration 2 mM, Sigma-Aldrich, St. Quentin Fallavier, France) to promote the formation of ethyl esters (Yee et al. 2014). Volatile compounds were extracted, analysed, and identified by headspace-gas chromatography-mass spectrometry (HS-GC-MS), using a TurboMatrix HS-40 trap as a headspace (HS) sampler, a Clarus 680 gas chromatograph coupled to Clarus 600T quadrupole mass spectrometer (Perkin Elmer, Courtaboeuf, France), as previously described (Pogacic et al. 2015). Briefly, samples of 2.5 g culture were placed in vials, they were warmed for 15 min at 65 °C, and the volatiles were extracted at a pressure at 207 kPa maintained in vial for 1 min with the carrier gas (helium), before being adsorbed on a Tenax™ trap at 35 °C. The trap load was repeated twice for each vial trap. Volatiles were then separated on an Elite 5MS capillary column (60 m × 0.25 mm × 1 μm; Perkin Elmer), with helium as the mobile phase. The initial temperature of the oven was 35 °C, maintained for 5 min. The oven temperature was then increased performed up to 140 °C at a rate of 7 °C.min^−1^ and then up to 280 °C at 13 °C.min^−1^. The mass spectrometer was operated in the scan mode, within a mass range of *m*/*z* 25–300, scan time 0.3 s, and interscan delay 0.03 s. Ionization was done by electronic impact at 70 eV. GC-MS data were processed as previously described (Pogacic et al. 2015), using the open source XCMS package implemented with the R statistical language (Smith et al., 2006), which converts the raw data to time- and mass-aligned chromatographic peaks areas. Volatile compounds were identified thanks to the mass spectral data Library NIST and to their retention index.

### Statistical analysis

An analysis of variance (ANOVA) was performed on the abundance of selected aroma compounds from triplicate cultures, using R statistical software, to determine if they significantly depend on the strains and on lac/nit phenotypes. Means were compared using the least significant difference (LSD) test. A principal component analysis (PCA) was performed on preprocessed, log10[*x*]-transformed and Pareto scaled data, using the package FactomineR of the software R.

## Results

### Lactose fermentation and nitrate reduction

Preliminary tests were performed on a pool of 15 strains to determine the effect of varying the concentrations of lactose and potassium nitrate on the results of the tests (data not shown). The results related to lactose fermentation were the same regardless of lactose concentrations. For nitrate reduction, one strain, *P. freudenreichii* B66, was detected positive at 0.5 g.L^−1^ potassium nitrate, but negative at 1.5 g.L^−1^. Therefore, the concentrations of 5 g.L^−1^ lactose and 0.5 g.L^−1^ nitrate were selected for further tests.

Table 1 summarizes the results of phenotype obtained for all the strains and the genotype determined after Loux et al. (2015) for the 10 sequenced strains. The results of lactose fermentation test were negative for the two strains that did not possess a complete lactose genomic island (CIRM-BIA121 and CIRM-BIA514) and positive for the eight strains which possess a lactose genomic island, as expected. The change in colour of bromocresol purple from purple to yellow (i.e. positive test) was clear only after 5 days of incubation, or even 7 days for CIRM-BIA516 and CIRM-BIA 135, and not after 2 days. For nitrate reductase, the five strains lacking a complete nitrate (*nar*) genomic island were effectively found negative regardless of the incubation time. The five strains that possess the *nar* genomic island showed a positive phenotype, after 2 days (three strains) or only 5 days of incubation (two strains, CIRM-BIA122 and CIRM-BIA513) (Table 1).

Out of the 18 Brazilian strains, nine showed the ability to ferment lactose, with five strains positive at 2 days and 4 strains only at 5 days. Seven strains were positive for nitrate reductase, with 3 and 4 strains detected positive after 2 and 5 days of incubation, respectively.

### Production of aroma compounds

Forty-two volatile compounds were detected in the headspace of the 28 cultures analysed (data not shown). Fifteen aroma compounds were selected among all the volatiles, based on several criteria: they are considered as important in the flavour of Swiss cheese, they result from different metabolic pathways, and they varied very significantly (*P* < 0.001) among strains (Table 2). They include compounds of diverse chemical nature (alcohols, esters, carbonyl compounds, aldehydes, acids, and sulphur-containing compounds).

**Table 2:** Selected volatile aroma compounds identified in cultures of *Propionibacterium freudenreichii* and *P* value of the ANOVA as function of the lac/nit phenotype and of the strain

RI	Compounds (trivial name)	Ion (*m*/*z*)^a^	Identification^b^	*P* value, lac/nit phenotype^cd^	*P* value, strain^d^
554	1-Propanol	59	RI,D	***	***
589	2,3-Butanedione (diacetyl)	86	S,RI,D	0.054	***
659	3-Methylbutanal	58	S,RI,D	***	***
666	2-Methylbutanal	57	S,RI,D	0.083	***
666	1-Butanol	56	RI,D	0.319	***
695	2,3-Pentanedione	100	RI,D	***	***
711	Ethyl propanoate	102	S,RI,D	0.697	***
712	2-Butanone-3-hydroxy (acetoin)	88	S,RI,D	*	***
739	3-Methylbutanol	55	S,RI,D	***	***
745	2-Methylbutanol	57	S,RI,D	*	***
752	Dimethyl disulphide	94	S,RI,D	0.370	***
809	Propyl propanoate	75	RI,D	***	***
845	2-Methylbutanoic acid	87	RI,D	***	***
872	3-Methylbutyl acetate	87	RI,D	**	***
910	Butyl propanoate	57	RI,D	**	***

*RI* Kovats retention index

^a^Ion used for quantification

^b^Compounds identified on the basis of S, retention time and mass spectrum from S, standard; RI, retention index; D, mass spectral data Library NIST

^c^lac/nit phenotypes: *freudenreichii*, *shermanii*, lac^+^/nit^+^, and lac^−^/nit^−^

^d^ *P* value of ANOVA: ***P < 0.001, **P < 0.01, *P < 0.05

**Fig. 1:**
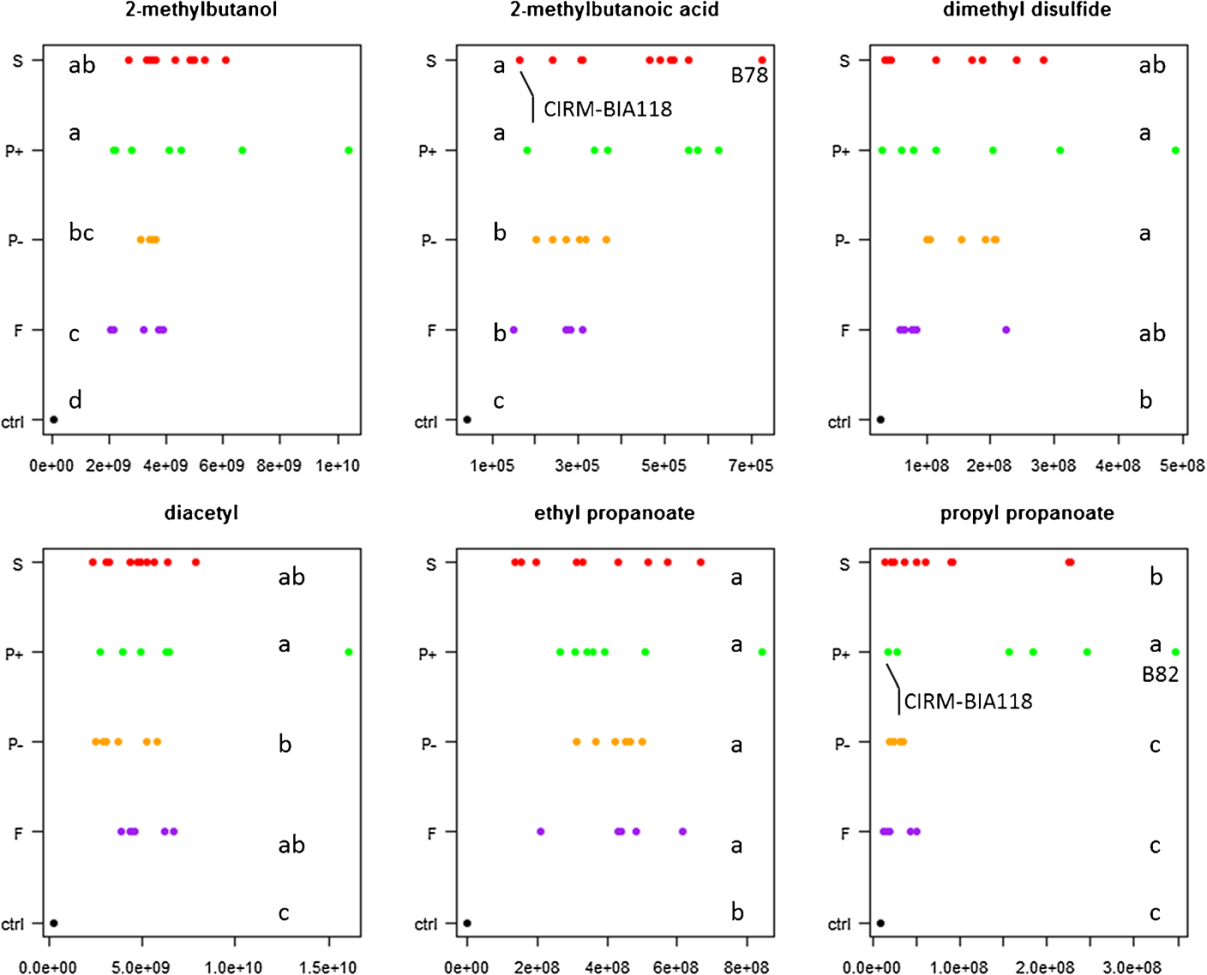
Abundance of six aroma compounds, expressed as area of a specific ion (*m*/*z*), in arbitrary units, in the cultures of 28 *P. freudenreichii* strains of four lac/nit phenotypes (*freudenreichii*, *shermanii*, lac^+^/nit^+^ and lac^−^/nit^−^), abbreviated as *F*, *S*, *P+* and *P−*, respectively). *ctrl*, control non-inoculated medium incubated under the same conditions; *a*–*d*, the production within phenotype with different letters differ significantly (*P* < 0.05)

**Fig. 2:**
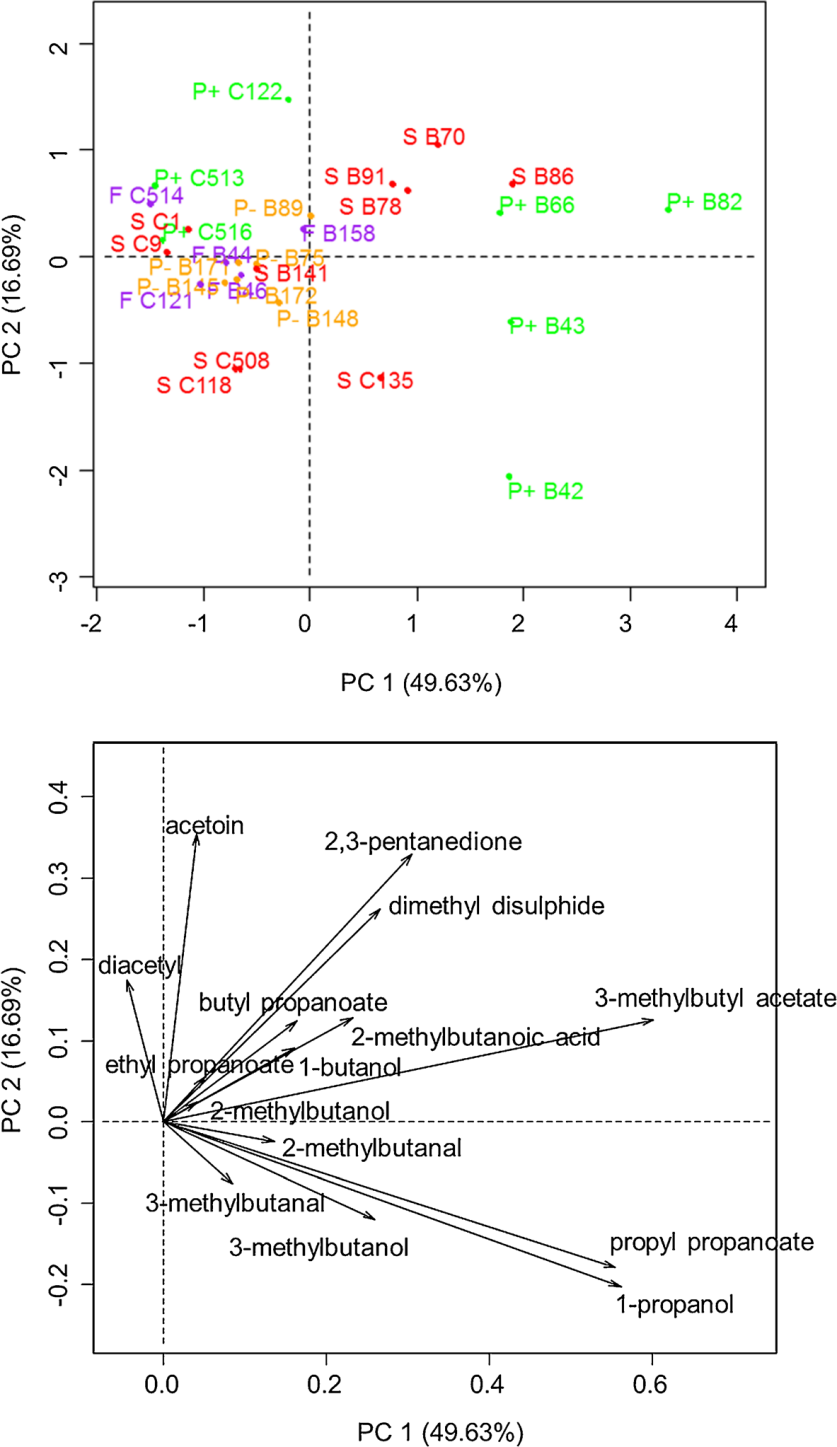
Results of principal component analysis (PCA) performed on 16 aroma compounds. PCA scores and loadings for the first two principal components. Cultures are encoded using the name of the lac/nit phenotype (*F*, *S*, *P+* and *P−*, for *freudenreichii*, *shermanii*, lac^+^/nit^+^ and lac^−^/nit^−^, respectively), followed by the name of strains (CIRM-BIA abbreviated as *C*)

The results show that the abundance of 10 of the 15 selected aroma compounds varied significantly between the four lac/nit phenotypes (*freudenreichii*, *shermanii*, lac^+^/nit^+^ and lac^−^/nit^−^) (Table 2). However, the variations between strains within lac/nit phenotypes were far greater that between lac/nit phenotypes, as illustrated for some compounds in Fig. 1. For example, the abundance of 2-methylbutanoic acid was 1.7-fold higher in lac^+^/nit^+^ (P+) and *shermanii* (S), on average, compared to *freudenreichii* (F) and lac^−^/nit^−^ (P−), but it showed still greater differences within the *shermanii* phenotype (up to 4.5-fold higher). For example, within *shermanii* strains, CIRM-BIA118 produced less 2-methylbutanoic acid than all other strains, whereas B78 was among the highest producing strains (Fig. 1). Similarly, the abundance of propyl propanoate was six-fold higher for strains of phenotype P+, on average, compared to F and P−. However, within the phenotype P+, the strains showed up to 20-fold differences for the production of this compound (B82 and CIRM-BIA513 with the highest and lowest production, respectively, Fig. 1). Other compounds, such as diacetyl, ethyl propanoate, and dimethyl disulphide, did not show any significant differences between lac/nit phenotypes (Table 2, Fig. 1). The abundance of the 15 aroma compounds was subjected to PCA to visualize the proximity of strains (Fig. 2). A cumulative variation of 66% was explained by the first two principal components. PC1-PC2 plot mainly differentiated the cultures on the basis of their content in esters, branched-chain compounds, and carbonyl compounds. The four phenotypes *freudenreichii*, *shermanii*, lac^+^/nit^+^, and lac^−^/nit^−^ appeared widespread on the map, showing that the groups are not related to the ability of producing any of the aroma compounds analysed.

## Discussion

This study was undertaken to re-examine the relevance of the subdivision of *P. freudenreichii* into two subspecies, which is currently based on two criteria, lactose fermentation, and nitrate reduction, and to investigate the significance of this subdivision to choose a strain as a culture for cheese manufacture.

Phenotypic methods are essential in the taxonomic characterization of microorganisms, but they are time-consuming and the results sometimes ambiguous. To give consistent results, phenotypic characterization should be applied by using strictly defined protocols adapted for each species, which are rarely available. In this study, we defined ad hoc conditions to determine lactose fermentation and nitrate reduction in *P. freudenreichii* by using 10 sequenced strains characterized for the presence or the absence of the corresponding genomic islands. The type strains of the two *P. freudenreichii* subspecies were also included. Our results show that a too short incubation time can lead to false nit^−^ results. Swart et al. (1998) showed that *Propionibacterium acidipropionici* and *P. freudenreichii* subsp. *freudenreichii* were only able to reduce measurable amounts of nitrate after 70 h of incubation, and, more globally, that the environmental conditions have a marked effect on the ability of propionibacteria strain to reduce nitrate. Therefore, difference in the incubation time could be responsible for the discrepancies observed between this study and previous reports for the same strains. Hence, three strains previously classified as *P. freudenreichii* subsp. s*hermanii* (lac^+^/nit^−^) CIRM-BIA122, 513, and 516 (Dalmasso et al. 2011) were identified here as nitrate reductase positive, in agreement with the phenotype expected from their genome. These three strains were thus reclassified as lac^+^/nit^+^ (P+, Table 1). Half of the strains characterized as nit^+^ in the present study actually showed a positive response only after 5 days of incubation. Our results also showed that a high concentration of potassium nitrate (1.5 g.L^−1^) in the test medium inhibited the growth of some strains, thus causing false nit^−^ results. These results illustrate how a lack of standardization of the experimental conditions can impact the results and in particular induce the detection of false negative traits, and demonstrate the importance of the integration of reference strains in taxonomic studies.

Many strains characterized in the present study could not be classified according to the currently defined subspecies. They were identified in both the CIRM-BIA collection and the pool of Brazilian wild strains. This result is in agreement with previous reports that occasionally mentioned the existence of strains exhibiting the phenotypes lac^+^/nit^+^ or lac^−^/nit^−^, in addition to the phenotypes corresponding to the two classical subspecies (Dalmasso et al. 2011, de Carvalho et al. 1994; Kaspar 1982; Moore and Holdeman 1974; Vorobjeva 1999). A subspecies called *P. freudenreichii* subsp. *globosum*, described as harbouring the phenotype lac^+^/nit^+^ (de Carvalho et al. 1994), was not maintained in the first edition of the *Bergey’s Manual of Systematic Bacteriology* (Cummins and Johnson 1986). Overall, 46% of the strains characterized in the present study could not be classified in one of the two subspecies of *P. freudenreichii*, with 25% of lac^+^/nit^+^ and 21% of lac^−^/nit^−^ strains. In comparison, 27% of the 113 strains characterized could not be classified into subspecies in the study of Dalmasso et al. (2011).

The four lac/nit phenotypes corresponding to the different combinations of lac/nit phenotypes were not distinguished by the production of any of the aroma compounds analysed. The aroma profile reflects the metabolic activity of the cells, since aroma compounds are synthesized via different pathways involving enzymes such as esterases and amino acid-converting enzymes (Abeijon Mukdsi et al. 2014, Thierry et al. 2002). The pathways of synthesis of these compounds are not related to the pathways of lactose fermentation or nitrate reduction, but these different phenotypic properties may nevertheless have been found correlated. The absence of relationship between the lac/nit phenotypes and other metabolic activities could be expected from the fact that the *P. freudenreichii* subspecies definition does not reflect the ancestral relationships between strains, as strongly suggested by several recent studies. Using multilocus sequence typing (MLST) applied to a large collection of *P. freudenreichii* strains, Dalmasso et al. (2011) showed that the strains were distributed randomly over the phylogenetic tree regardless of their lac/nit phenotypes. Moreover, the data acquired on the genome sequence of *P. freudenreichii* CIRM-BIA1^T^ highlighted new insights on the origin of the random phenotypical variations concerning lactose fermentation and nitrate reduction in this species (Falentin et al. 2010). The lactose genomic island of this strain is surrounded by integrases and transposases, suggesting that the *lac* genes may have been acquired through horizontal transfer, whereas the gene corresponding to the β-subunit of nitrate reductase (*narH*) is a pseudo-gene due to a frameshift. Similar mechanisms may explain the very diverse phenotypes observed concerning the production of aroma compounds. The different strains tested did not group together, but rather exhibited a continuum in their ability to produce aroma compounds, as previously observed for 2-methylbutanoic acid (Dherbécourt et al. 2008) and also for free fatty acids coming from lipolysis (Abeijon Mukdsi et al. 2014).

## Conclusion

This study confirms that a very large proportion of strains cannot be classified into the two currently defined subspecies of *P. freudenreichii* and demonstrates that the ability to ferment lactose and reduce nitrate are not related to each other in *P. freudenreichii*, thus generating four phenotypes based on these two criteria, and not two. Moreover, the results of this study show that belonging to a lac/nit phenotype cannot give any idea of the ability of a *P. freudenreichii* strain to produce any of the important aroma compounds analysed. The choice of a strain for a specific contribution in a given cheese technology should thus be done according to its specific technological properties. Taken together with some other recent studies, these results strongly suggest that maintaining the division of *P. freudenreichii* into two subspecies does not appear to be relevant.
